# Mood State Changes Accompanying the Crossfit Open™ Competition in Healthy Adults

**DOI:** 10.3390/sports6030067

**Published:** 2018-07-23

**Authors:** Allyson G. Box, Yuri Feito, Steven J. Petruzzello, Gerald T. Mangine

**Affiliations:** 1 Department of Kinesiology and Community Health, University of Illinois at Urbana-Champaign, 906 S Goodwin Ave, Urbana, IL 61801, USA; box.allyson@gmail.com (A.G.B.); petruzze@illinois.edu (S.J.P.); 2 Department of Exercise Science and Sport Management, Kennesaw State University, 520 Parliament Garden Way NW, Kennesaw, GA 30144, USA; gmangine@kennesaw.edu

**Keywords:** fatigue, energy, exercise, high-intensity functional training

## Abstract

Background: Specific mood states were examined during the CrossFit Open, a consecutive 5-week fitness competition involving five separate CrossFit^®^ workouts, to determine whether the unique design or strenuous workouts of the competition resulted in acute and/or chronic mood state alterations. Methods: Participants (*n* = 8) completed the Profile of Mood States (POMS) questionnaire one-week prior to the competition (baseline), prior to (PRE), immediately post (IP), 30-min post- (30P) and 60-min post-workout (60P) each week. Tension, depression, anger, confusion, fatigue and vigor were derived from the POMS, as was Total Mood Disturbance (TMD) and an Energy Index (EI). Results: Workout intensity exceeded 93% HR_max_ each week. No differences were observed between baseline and PRE-workout mood states across weeks, indicating little effect of the unique competition design. Significant (week x time) interactions were observed for TMD (*p* = 0.037), EI (*p* = 0.038) and fatigue (*p* = 0.005). Acute mood state fluctuations were consistent across each week, where mood states improved to and beyond PRE values 60-min post-workout. Conclusions: In competitors, the differences in workout design between each week did not influence mood states. This may be related to adaptation to this style of training, while the acute mood state alterations are likely due to the workout intensity.

## 1. Introduction

Affective responses (e.g., core affect, emotions and mood states) are known to be influenced by the modality and intensity of an exercise session [1,2]. Simply, core affect is defined as a general, fluid feeling that is adaptive in nature that can influence emotion and mood and vice versa. Emotions tend to be more intense, disperse more quickly and can be directly attributed to a stimulus, while a mood state is defined as a less intense, longer diurnal state that may or may not be directly attributed to a specific stimulus [3]. The Circumplex model is a commonly used theoretical framework for assessing affective fluctuations in physical activity and exercise [4]. Briefly, the Circumplex is thought to express the broad dimensions associated with core affect (i.e., an individual’s most general and basic feelings) using a two-dimensional structure (i.e., high/low activation & pleasant/unpleasant feelings) [5]. Broad dimensional changes reflect the subtle changes in how one is feeling and can result from shifts in feeling generally pleasant to unpleasant, activated (energetic) to deactivated (calm), or vice versa. Although basic and general feelings (e.g., valence & activation) do not represent specific emotions or moods, researchers have associated emotions to the Circumplex [5]. Thayer (1986) suggested there are four quadrants comprising the valence/activation continuum: High activation-pleasant (e.g., vigor, energy), high activation-unpleasant (e.g., tension), low activation-unpleasant (e.g., tiredness) and low activation-pleasant (e.g., calmness). These four quadrants result from a 45 degree right-shift from the original Circumplex [6]. Although the Circumplex model of affect is not domain specific and typically refers to “global affective states” that couple pleasant or unpleasant feelings and feelings of activation [5], some specific emotional/mood domains (i.e., vigor, tension) can be associated to this model.

When examining affective responses to exercise, researchers have generally found that as intensity increases, there is a progressive alteration from positive to negative affect [1,7,8,9]. Specifically, higher-intensity exercise (e.g., 85% HRR, 80–85% age predicted HR_max_) has been reported to increase measures of tension, fatigue and unpleasant affective responses during and immediately following exercise [1,2,10]. However, these negative states typically rebound (i.e., become less negative or become positive) during the recovery period (e.g., 10–90 min post-exercise) [1,2,7]. Specifically, Hall and colleagues [10] reported improvements in affective valence, using the Feeling Scale, beyond pre-exercise values as early as 10-min following an exercise session (i.e., graded exercise test), even though this session resulted in predominately negative feelings and an average Rating of Perceived Exertion of 17.8 (defined as “Very Hard”) while exercising.

Although high intensity training has been shown to increase negative feelings during exercise (i.e., tension/anxiety), individuals report improved feelings of energy and enjoyment after completion of high intensity exercise [1,11,12,13]. In contrast, the impact of exercise duration on mood states is not as clear. Hansen, Stevens and Coast [14] reported that continuous, longer-duration exercise (i.e., 30 min) resulted in greater feelings of fatigue compared to a 10-min bout of exercise (intensities were the same for the different durations). Beyond that study, however, no evidence exists to suggest an impact on mood from the duration of exercises. Nevertheless, these data suggest that higher intensity and longer duration exercise elicits greater responses of specific negative mood states (i.e., tension, fatigue) or more unpleasant affective responses during and immediately following exercise. Further, the negative post-exercise response is improved during the recovery period.

According to the American College of Sport Medicine annual survey on worldwide fitness trends, high-intensity training has been a top fitness trend for the last 10-years and hit the top spot for 2018 [14]. CrossFit^®^ Training (CFT), one prominent example of high-intensity training, uses “constantly varied, high-intensity, functional movements” to achieve general physical preparedness [15]. A possible explanation for the upsurge in popularity of CFT may be related to the exercise programming and social aspects that are unique to CFT [16,17]. CFT programming (i.e., exercise intensity, duration, organization and complexity) varies by day and location but also contains several common elements. Workouts are typically performed in a group setting and designed to be either completed as quickly as possible, or they require participants to complete “as many repetitions as possible” (AMRAP) within a given time frame. Since individual pace and effort within every workout is self-selected [18], this training strategy appears to accommodate both novice and advanced individuals. Indeed, novice and experienced participants report that the CFT culture (e.g., gym appearance, common member goals of improving health) and social nature of member interactions are primary factors for their continued involvement [16,19], while more frequent CFT participation has been observed to produce greater feelings of basic need satisfaction [20]. In turn, evidence suggests that regular exercisers report more positive affect and less anxiety immediately following a vigorous exercise bout [21]. However, the effects of CFT on mood (or affect) remains unknown.

The CrossFit^®^ Games is an annual competition that seeks to find “the fittest on earth^®^”. The preliminary round of this competition, the CrossFit Open™ (CFO), is a worldwide event open to anyone and it consists of five separate workout challenges spread across five consecutive weeks. Each challenge is unique and unknown to the competitors prior to its release, although they typically include a combination of specific CFT movements. Upon each workout’s release (Thursday, 5:00 p.m. PST), competitors are allotted four days to complete the event and submit their best performance result to competition officials (by Monday, 5:00 p.m. PST). From the initial pool of competitors (*N* = 324,307 in 2016), only 640 participants continue to the next level of competitions. With such a small percentage (0.2%) of initial competitors ultimately progressing to the next round (i.e., Regionals), objective success may not be the sole motivational factor contributing to this contest’s popularity. As the majority of individuals (>99%) completing the CFO are not “elite” competitors, it is possible self-referenced performance criterion (e.g., percentage of personal best, achievement of performance goals, intra-affiliate/gym rivalries) or affective states (e.g., positive affect, enjoyment) contribute towards individual involvement in CFT and the CFO.

Successful performance (objective or self-referenced) has been suggested to be influenced by mood [22,23]. In fact, small-to-moderate effect sizes have been reported between the Profile of Mood States (POMS) questionnaire subscales and sports performance (0.13–0.47), particularly when success was based on self-referenced criteria compared to objective measures (e.g., ranking, winning-or-losing; [22]). A greater effect was also observed in short-duration (<10 min), unpredictable sports [22], though this has been argued to the contrary [23]. However, the CFO is difficult to classify as a sport. Although workout design may vary, competitors may still rely on several familiar elements being present (e.g., specific exercises, workout structures, at least one previous CFO event is usually repeated) and they are also given the option of completing the workouts “as prescribed” (i.e., Rx) or in a less difficult, scaled way. Thus, the CFO’s adaptability may be indicative of both objective and self-referenced indicators of success being present. Further, because the CFO occurs over several weeks, mood may vary between events, which is contrary to the consistency observed between competitions in traditional team sports [24,25].

Therefore, the purpose of this investigation was to observe whether any changes in six specific mood states occurred across a 5-week CFO competition or in response to each individual workout challenge. We hypothesized that specific mood state changes would occur within each weekly competitive bout (i.e., increased negative mood) and subside during the recovery period immediately following exercise. Further, we postulated that no significant differences would occur between weeks in a group of relatively experienced participants. To our knowledge, this is the first investigation to examine mood state responses surrounding a CFT competition.

## 2. Materials and Methods

### 2.1. Participants

Twelve male (*n* = 5) and female (*n* = 7) adults with more than 6-months of CFT experience, who had signed-up for the 2016 CrossFit Open^TM^ prior to recruitment, were enrolled in this investigation. These participants were recruited from a local CrossFit^®^ affiliate. Following an explanation of all procedures, risks and benefits, each participant provided written informed consent to participate in the study. All participants were free of any known contraindications to moderate or high intensity exercise (ACSM, 2013) and did not have any musculoskeletal, cardiovascular, pulmonary, or metabolic conditions that limited their ability to exercise (determined from medical history questionnaire); females who were pregnant were excluded. Over the course of the 5-week study, eight of the original 12 participants completed all aspects of data collection (34.3 ± 5.6 years, 77.8 ± 16.2 kg, 164.7 ± 10.1 cm). Of the four who did not complete the study, one cited job-related circumstances and the other three cited personal reasons. Data were only analyzed from the participants who completed all aspects of the study (Table 1). The institution’s Ethics Review Board approved all procedures and study protocols prior to any enrollment of participants in the study.

### 2.2. Experimental Design

The study protocol was divided into six individual sessions, with each testing session taking place one week apart. The first testing session (BL) took place at the university’s exercise physiology laboratory, while all subsequent sessions took place at a local CrossFit^®^ affiliate (gym). During the BL visit, potential participants were familiarized with the study’s purpose, risks and benefits and then completed all enrollment documents (i.e., medical history, activity questionnaire, physical activity readiness questionnaire and written informed consent). Subsequently, enrolled participants completed all BL assessments. During succeeding weeks (Weeks 1–5), all participants wore a HR monitor to assess relative intensity and completed the mood assessment prior to and following the workout. The study was concluded following all post-exercise assessments on the last week (Week 5) of the competition.

### 2.3. Baseline Assessments

Following enrollment, baseline anthropometric, resting heart rate and mood assessments were collected. Height (±0.1 cm) and body mass (±0.1 kg) were recorded using an electronic physician’s scale (Tanita WB 3000, Arlington Height, IL, USA) with participants wearing light and comfortable clothing (e.g., shorts, t-shirt). Following all anthropometric assessments, participants were fitted with a heart rate (HR) monitor (Polar Team 2, Lake Success, NY, USA) and asked to remain seated in a dark, quiet room for 10 min to determine an average resting HR. Immediately following resting HR assessment, the participants were asked to complete the Profile of Mood States (POMS) questionnaire (26).

### 2.4. Competition Sessions

The 5-week online competition was comprised of a single workout each week for 5 consecutive weeks (Week 1 = 16.1, Week 2 = 16.2, Week 3 = 16.3, Week 4 = 16.4, Week 5 = 16.5). Each week’s workout was released on Thursday evening at 5:00 p.m. PT and competitors were allotted 4-days to submit their best score by the following Monday (at 5:00 p.m. PT) to remain in the competition. Competitors were not aware of any of workout details prior to its release but were allowed unlimited attempts (within the 4-days time span) to achieve the best possible score. As such, for this study testing was completed on the first attempt in order to better examine the effects of the unique competition design, as each workout release is unknown and participants are unlikely to optimally strategize before the first attempt. The composition of each of the five workouts is described in Table 2.

All testing occurred at a consistent time of day (12:00–3:00 p.m.) on a Friday or Saturday during the first four weeks of the competition. During week 5, however, all participants completed the workout on the night of its release (Thursday) instead of their regular scheduled time. On each week of the competition, the participants would arrive at their local CrossFit^®^ affiliate (gym) approximately 30–60 min prior to their scheduled workout time slot.

Participants were fitted with a HR monitor and asked to complete the POMS prior to (PRE) the workout following a 10-min rest period. After completing the questionnaire and before completing the workout of the week, each participant completed a self-selected warm-up. The investigators did not have any influence on the warm-up period for any of the participants. Upon completing the workout, the participants were asked to complete the POMS immediately post- (IP), 30 min post- (30P) and 60 min post-workout (60P). All testing was completed within the local CrossFit^®^ affiliate’s “box” (i.e., an open gym space).

### 2.5. Questionnaire

A modified 58-item unipolar Profile of Mood States (POMS; [26]) questionnaire, rather than the original 65-item POMS [27], was used to assess several distinct mood states. The POMS was used to assess differences in specific mood states that occurred across multiple weeks, as well as fluctuations that may occur across the time span surrounding competitive workouts. The modified POMS is comprised of six distinct mood subscales (7–15 items per subscale; Chronbach’s *α* = 0.63–0.93): Vigor-activity (Vigor), Fatigue-inertia (Fatigue), Tension-anxiety (Tension), Depression-dejection (Depression), Confusion-bewilderment (Confusion) and Anger-hostility (Anger). A seventh subscale, Friendliness and its subsequent 7-items (i.e., friendly, clear-headed, considerate, sympathetic, helpful, good-natured and trusting) from the original POMS, were removed due to reports in lack of reliability [27,28]. Participants were asked to respond to each item using a 5-point Likert-Scale (0 = “Not at all”, 1 = “A little”, 2 = “Moderately”, 3 = “Quite a bit”, or 4 = “Extremely”) based on how they felt “right now”, rather than recalling how they felt in the past week or even the past 24 h. The values of items associated with a specific subscale (e.g., Vigor) were summed to calculate the score for that specific subscale. Additionally, Total Mood Disturbance (TMD) and Energy Index (EI) scores were calculated. The TMD was calculated by subtracting the Vigor score from the sum of the other five negative mood subscales and adding 100 as a constant (score range: 68–268). In this way, a higher TMD score is indicative of greater mood disturbance (i.e., greater negative mood). Further, an EI score was calculated because previous research has noted that the Vigor and Fatigue subscales of the POMS are most responsive to acute exercise bouts [29,30]. The EI score was calculated by subtracting Fatigue from Vigor to reflect a participant’s overall perceived energy [31,32]. As such, a more negative EI score would be indicative of lower vigor and/or greater fatigue, whereas a more positive EI score indicates greater vigor and/or less fatigue (score range: −28–+32). The modified POMS has been shown to be a valid and reliable psychometric tool to assess an individual’s mood [26].

### 2.6. Exercise Intensity and Duration

For each week, a HR monitor was worn from rest to 30 min post-workout to provide a measure of relative workout intensity. Each participant’s maximal heart rate (MHR) was estimated using the equation “220 − Age (in years)” [33] and then divided into their average heart rate during each week’s workout to calculate percent of maximum heart rate (%MHR). The relative intensities and workout durations (in minutes) for each participant during each workout of the 5-week competition are presented in Table 3.

### 2.7. Statistical Analysis

Separate one-way analyses of variance with repeated measures (RMANOVA) were performed to examine differences in %MHR and workout duration, as well as differences between BL and PRE-measures of TMD, EI and POMS subscales, across the 5-week competition. Following a significant *F*-ratio, specific differences between weeks were assessed using the least-significant difference (LSD) post-hoc analysis procedure.

To determine the effect of the workout on changes in mood, separate two-way RMANOVAs were performed on each dependent variable, where Week (Week 1, Week 2, Week 3, Week 4 and Week 5) and Time (PRE, IP, 30P and 60P) were included as the main factors. Where violations of the sphericity assumption occurred, the Huynh-Feldt epsilon (H-F ε) was used to adjust degrees of freedom. Significant main effects, interactions and post-hoc analyses were assessed using Least Significant Difference (LSD) procedure. Additionally, effect sizes are reported as partial eta^2^ (*η^2^_p_*) for ANOVAs for comparison across weeks or time. For *η^2^_p_*, values of 0.01, 0.06 and 0.14 are interpreted as small, moderate and large effect sizes, respectively [34]. SPSS statistical software (SPSS, v.22, Chicago, IL, USA) was used for all analyses with statistical significance set at *p* < 0.05. All data are reported as a mean (*M*) ± standard deviation (*SD*).

## 3. Results

### 3.1. Workout Intensity and Duration

A significant difference between competition weeks for %MHR (*F*(2.3, 16.4) = 4.53, *p* = 0.023, *η^2^_p_* = 0.39; H-F ε = 0.59) was observed, where %MHR for Week 5 (96.96 ± 6.45%) was significantly higher than any other week (see Table 3; pairwise comparisons *p_s_* ≤ 0.022, *d*_s_ = 0.34–0.55). Significant week differences were also observed for workout duration (*F*(2.6, 18.5) = 38.69, *p* < 0.001; H-F ε = 0.66). Specifically, Week 1 (20 min) and Week 5 (17.6 ± 4.5 min) were significantly longer (*p* ≤ 0.02) in duration compared to Week 2 (8.5 ± 3.7 min), Week 3 (7 min) and Week 4 (13 min). Further, Week 2 and Week 3 were significantly shorter (*p* ≤ 0.015) in duration than all other weeks but not different from each other (*p* = 0.32). No other differences between weeks for %MHR or workout duration were found.

### 3.2. Baseline Comparisons

No significant differences were observed between BL and PRE-scores during any week of the competition for any measurement obtained from the POMS. All BL and PRE-scores for the individual POMS subscales, TMD and EI are presented in Table 4.

### 3.3. POMS Subscales

**Figure 1:**
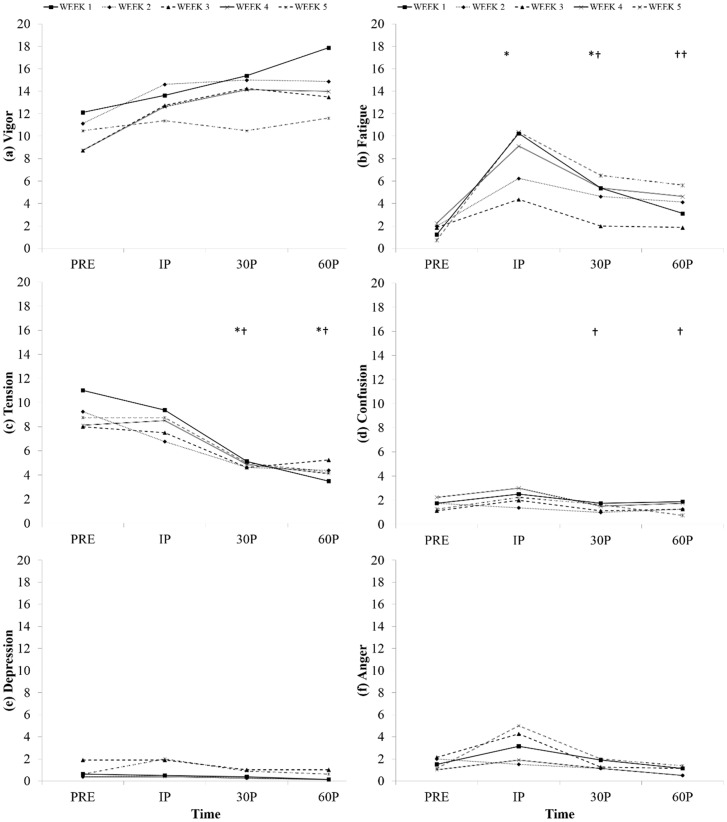
Time changes in POMS subscales from PRE to Immediately Post (IP), 30-min Post (30P) and 60-min Post (60P): Time changes in (**a**) Vigor; (**b**) Fatigue; (**c**) Tension; (**d**) Confusion; (**e**) Depression; (**f**) Anger. * Indicates a significant difference from PRE, ^†^ from IP at a *p* < 0.05.

For the six distinct mood states, a significant Week x Time interaction was observed only for Fatigue (*F*(9.1, 63.8) = 2.96, *p* = 0.005, *η^2^_p_* = 0.297, H-F ε = 0.76; see Figure 1). When compared to PRE (W1: 1.25 ± 2.55; W4: 2.25 ± 5.26; W5: 0.75 ± 1.04), a significant increase was observed at IP during Week 1 (10.25 ± 4.03, *p* = 0.001), Week 4 (9.13 ± 5.87, *p* = 0.001) and Week 5 (10.38 ± 7.29, *p* = 0.007). Although Fatigue scores returned to pre-exercise values at 30P and 60P for Week 1 (5.34 ± 6.57, 3.13 ± 5.17; respectively, *p* > 0.05), Fatigue values did not return to pre-exercise values for Weeks 4 (30P: 5.38 ± 6.48, *p* = 0.014; 60P: 4.63 ± 5.68, *p* = 0.034) or 5 (30P: 6.50 ± 5.95, *p* = 0.032; 60P: 5.63 ± 4.96, *p* = 0.031). In comparing weeks, Fatigue differences were observed at IP where Week 3 (4.38 ± 5.21) was significantly lower compared to Weeks 1 (10.25 ± 4.03, *p* = 0.005), 4 (9.13 ± 5.87, *p* = 0.001) and 5 (10.38 ± 7.29, *p* = 0.008). While no other significant interactions were observed for any other subscale, a significant (*p* < 0.001) main effect for Time was found in Tension scores (see Figure 1). Specifically, Tension scores were significantly less at 30P (*p* = 0.003) and 60P (*p* = 0.002) when compared to PRE-scores during each week.

### 3.4. Total Mood Disturbance (TMD)

**Figure 2:**
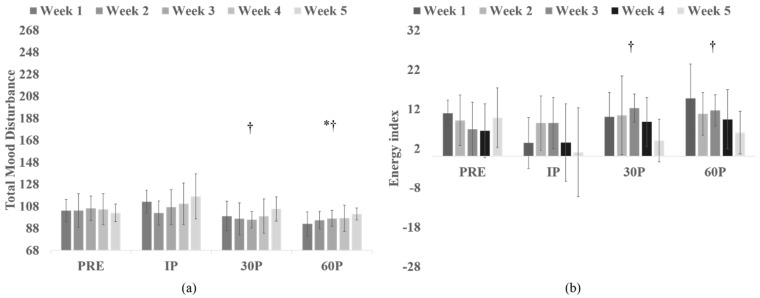
(**a**) Time changes in Total Mood Disturbance from PRE to Immediately Post (IP), 30-min Post (30P) and 60-min Post (60P); (**b**) Time changes in Energy Index from PRE to Immediately Post (IP), 30-min Post (30P) and 60-min Post (60P). * indicates significant difference from PRE, ^†^ from IP.

A significant Week x Time interaction was observed for TMD (*F*(7.0, 48.8) = 2.36, *p* = 0.037, *η^2^_p_ =* 0.25, H-F ε = 0.58; Figure 2a) where significant main effects for time were observed during Week 1 (*p* < 0.001), Week 3 (*p* = 0.023), Week 4 (*p* = 0.004) and Week 5 (*p* = 0.029). Compared to PRE-scores, TMD on Week 1 was significantly elevated at IP (*p* = 0.042) and then significantly lower at 30P (*p* = 0.046) and 60P (*p* = 0.012). Likewise, reduced TMD scores (from PRE) were observed at 30P (*p* < 0.03) and 60P (*p* < 0.02) on Weeks 3 and 4. During Week 5, TMD scores tended (*p* = 0.064) to be elevated from PRE-scores at IP. Across weeks, significant differences between weeks were noted at 30P (*p* = 0.037) where TMD scores were significantly higher on Week 5 (105.5 ± 11.0) compared to Week 1 (99.1 ± 13.4, *p* = 0.039), Week 2 (96.6 ± 14.6, *p* = 0.043) and Week 3 (95.8 ± 7.4, *p* = 0.014). No other differences in TMD scores were observed. Differences in TMD scores within and across each week are presented in Table 5.

### 3.5. Energy Index (EI)

In examining EI, a significant Week x Time interaction (*F*(9.7, 68.0) = 2.10, *p* = 0.038, *η^2^_p_ =* 0.23, H-F ε = 0.81; Figure 2b), as well as significant main effects for Week (*F*(4, 28) = 2.88, *p* = 0.041, *η^2^_p_* = 0.29) and Time (*F*(3, 21) = 3.09, *p* = 0.049, *η^2^_p_* = 0.31) were seen (see Table 5). Time main effects revealed differences only for Week 1, where PRE-scores (10.88 ± 3.36) were significantly greater than IP-scores (3.38 ± 6.50, *p* = 0.012). In comparing week effects at 30P, Week 5 (4.00 ± 5.42) scores were significantly lower than Week 3 (12.25 ± 3.58, *p* = 0.003). Further, at 60P, Week 5 (6.00 ± 5.40) scores were significantly lower than Weeks 1 (14.75 ± 8.66, *p* = 0.005) and 3 (11.63 ± 4.00, *p* = 0.005).

## 4. Discussion

The primary purpose of this study was to examine whether mood states changed throughout five-weeks of The CrossFit^®^ Open. Our findings suggest that consecutive weeks of this competition did not result in any significant changes in six specific mood states prior to each weekly workout in this group of participants. These findings are consistent with previous research, where little change in feeling states have been seen prior to sport competitions occurring across several weeks [24,25]. Specifically, Lane and Chappell [25] did not observe substantial mood changes prior to multiple basketball games that occurred on different days. Similarly, Alix-Sy, Le Scanff and Filaire [24] found that affect (pleasantness, unpleasantness) remained similar prior to different soccer competitions. However, it should be noted that these previous studies examined consecutive matches of sport competition, which differed from this study in that for a typical sport match, duration, rules and expectations are known and standard, whereas the CFO was comprised of different duration workouts and included different movements in each week. Further, it is assumed that CFO participants have little time to strategize before competing, which again, differs from a traditional sport (i.e., basketball, soccer). Regardless of these competition differences, the lack of change in mood states prior to exercise across the different weeks of the CFO in the present study could indicate that the unique design of the competition had little effect on pre-workout mood states.

The acute changes in mood states we observed following each individual exercise bout and their subsequent return to pre-workout levels during the 60-min recovery period, was consistent with our hypothesis and previous investigations where negative mood states (i.e., fatigue, TMD) increased immediately following the exercise and returned to and beyond pre-exercise values during the recovery period [7,9,35]. Feelings of Fatigue and TMD, or a decrease in affect (i.e., decreased pleasantness/increased unpleasantness), can be expected during and immediately following high-intensity exercise due to the physiological demands required to perform the activity [4]. In contrast to the findings of Herring and O’Connor [36], Vigor, the single positive mood state, remained consistent from pre- to post workout for each week of this study. As such, the changes seen in the Energy Index (EI) were mainly the result of the acute changes in Fatigue scores. The workouts with the greatest intensity coupled with the longest durations (Weeks 1 & 5) resulted in the highest levels of reported post-workout Fatigue and thus the lowest EI scores. This leads us to believe that both the intensity and duration of a workout within the CrossFit Open appear to be the likely factors that modulate the post-workout mood response.

To our knowledge, changes in mood and affect during and following acute bouts of CFT have not been documented. Our findings represent a first look at how mood response is affected by this type of high intensity, competitive exercise. Nonetheless, these findings are supported by previous studies reporting similar changes in mood during [1,2] and following high-intensity exercise [7,36]. A more negative mood response is expected to be elicited during high intensity exercise. Hall, Ekkekakis and Petruzzello [7] reported a marked decrease in positive affect in conjunction with an increase in negative affect as exercise approached ventilatory threshold during a bout of high intensity running. However, after the cessation of exercise, the authors observed substantial increases in positive affect. Similarly, Herring and O’Connor [36] reported increased vigor and decreased fatigue for up to 60 min following a resistance training program of different intensities (15 and 70% of 1RM). Further, the duration of an exercise bout appears to influence the responsiveness of specific mood states. Even though previous studies examining mood and affective responses to different exercise durations have reported mixed results on pleasantness and/or arousal, generally, longer duration exercise elicits an increasingly less positive affective response. Ekkekakis and Petruzzello [2] suggested that affective response could also be influenced by the participant’s fitness status. However, this is difficult to generalize considering studies have utilized different methods to address intensity [2]. In the present study, each exercise bout lasted less than 30 min and the shortest duration bout (i.e., Week 3; 7 min) resulted in less fatigue at IP (4.38 ± 5.21) compared to the longest duration bout at IP (i.e., Week 1; 20 min; 10.25 ± 4.03). Interestingly, the shortest duration workout also resulted in lowest Vigor scores when compared to the other weeks. Thus, among bouts of high-intensity exercise of different duration, longer duration bouts appear to have a greater effect on subsequent alterations in specific mood states.

The present investigation focused on mood surrounding the five workouts of the CrossFit Open. Although these workouts were part of a competitive event, these events are more scalable than those included in subsequent rounds (i.e., regionals and The Games^TM^). This helps to ensure that most affiliates are suitably-equipped to accommodate the various challenges and thus, it enables individuals of all fitness levels and locations to participate. In general, positive improvements in post-exercise mood (i.e., decreased tension, fatigue, TMD) were observed in the present study. These improvements may explain the growing popularity of high-intensity fitness programs [14] and their association with potentially stronger adherence rates. Heinrich and colleagues [37] alluded to greater rates of adherence intention among individuals who completed a CrossFit style program compared to those who completed a traditional aerobic and resistance training program. Williams and colleagues [38], examining affective responses during moderate intensity exercise, found that those who reported more positive affect (i.e., greater pleasantness) were more likely to increase their physical activity 6- and 12-months after the acute exercise bout. Moreover, Bartlett and colleagues [8] found that recreational athletes who ran high intensity intervals experienced more enjoyment than those who ran at a continuous pace, even though they were working at a higher rate of perceived exertion. CFT may result in similar responses in enjoyment and may be a good model for individuals to meet and adhere to current exercise recommendations.

### Limitations

A potential confound to our findings may be related to the consistency between weeks for completing the events. That is, the participants completed the first four challenges on a separate day (during the day) following its release (i.e., at least 12 h post-release), whereas the final week’s challenge was completed at night, within one hour of its release. It is possible that differences in the time of day and preparation time could have been responsible for the specific mood differences in TMD and EI observed during Week 5 compared to other weeks. A previous study examining mood state responses during different periods of the circadian rhythm found fluctuations in mood subscales, specifically Tension, Depression, Anger, Fatigue and Confusion all declined, while Vigor increased, from morning to night [39]. For our study, pre-workout values were similar across all five weeks compared to baseline values, which suggests that prior knowledge of the workout did not have any undue influence on mood. However, even though pre-workout values were similar across the competition, there were some differences immediately post workout and into recovery during Week 5 compared to the other weeks of the competition. 

Other potential limitations of this investigation include the small sample size and the use of a single psychometric tool (i.e., the POMS) to assess mood. Although the POMS questionnaire has been deemed valid and reliable for measuring mood [26,40], it was initially developed to assess clinical psychiatric patients [41]. It also does not assess the general mood domain. Rather, it assesses six distinct mood states that are negatively (mood) biased (i.e., Vigor is the only positive mood subscale) [3]. This has led some to question whether the POMS is suitable for measuring affective responses to exercise [42]. Further, the POMS lacks a theoretical foundation and does not assess affect. Although the Circumplex model is a suitable theoretical foundation for understanding affective responses, it is not the best model for domain-specific responses [7]. However, as we were interested in examining specific mood states (i.e., vigor, fatigue, tension) that resulted from a competition setting, the POMS questionnaire was our tool of choice. Our findings may have limited relation to the generalization of core affect and therefore, should only be interpreted as specific mood state changes rather than affective responses. Future research should incorporate additional psychometric questionnaires (i.e., measures of affective states) to gain a better understanding of affective responses during a CFT exercise bout.

## 5. Conclusions

Our findings are a first attempt to gain a better understanding of the changes in mood response that result immediately and for up to an hour following a competitive bout of CrossFit^®^ training. Our data suggests that baseline mood (in a controlled setting) and pre-workout mood did not differ across the 5-weeks of the competition, nor did the five bouts chronically alter mood. Even though certain negative moods increased immediately following the workout (i.e., Fatigue), these values declined to pre-workout values during recovery, while feelings of Vigor remained elevated throughout the entire session. This suggests that competitive bouts of CrossFit^®^ Training result in a positive effect on mood. Future studies focusing on mood response during a CFT session are encouraged to gather additional psychological variables (e.g., affective responses during competition, enjoyment, satisfaction in performance) and control for environmental and social confounds, which may elicit changing mood response.

## Figures and Tables

**Table 1:** Descriptive Information for the Participants.

	Age	Sex	Height (cm)	Weight (kg)	BMI	% Total Fat	Overall CFO Rank
	26.0	Female	167.1	74.2	26.6	18.7	
	38.0	Female	159.3	84.7	33.4	32.1	
	35.0	Female	161.8	103.1	39.4	44.4	
	31.0	Male	177.8	76.8	24.3	10.1	
	35.0	Male	166.9	80.9	29.0	13.4	
	33.0	Male	177.8	79.6	25.2	13.4	
	45.0	Female	158.8	78.5	31.1	33.1	
	31.0	Female	147.8	44.2	20.2	18.8	
Average	34.3 ± 5.6		164.7 ± 10.1	77.8 ± 16.2	28.7 ± 6.0	23.0 ± 12.1	40,508.6

CFO Rank refers to where the participant ranked out of the 340,000+ individual who took part in the CFO.

**Table 2:** Competition Workout Descriptions.

		Week 1	Week 2	Week 3	Week 4	Week 5
Workout Description (One round equals the completion of the designated movements)		As Many Repetitions as Possible (AMRAP) in 20 min.	4-min rounds Must complete designated movements within each round to move into next round.	7-min AMRAP	13-min AMRAP	Complete descending repetition scheme (21-18-15-12-9-6-3 each) for time
Reps/Movement/Weight (Male/Female)	**Rx**	25-ft Overhead Walking Lunge (43 kg/29 kg) & 8 Burpees & 25-ft Overhead Walking Lunge (43 kg/29 kg) & 8 Chest-to-bar pull-up	25 Toes-to-bar & 50 Double-unders & Squat Clean ° (15-13-11-9-7 rep scheme)	10 Power Snatches (34 kg/25 kg) & 3 Bar Muscle-ups	55 Deadlift (102 kg/70 kg) & 55 Wall-ball Shot * & Row (55 calories) & 55 Handstand Push-Up	Thruster (43 kg/29 kg) & Burpees
	**Scale**	25-ft Front Rack Walking Lunge (29 kg/15 kg) & 8 Burpees & 25-ft Front Rack Walking Lunge (29 kg/15 kg) & 8 Jumping Chin-Over-Bar pull-up	25 Hanging Knee Raises & 50 Single-unders & Squat Clean ^◊^ (15-13-11-9-7 rep scheme)	10 Power Snatches (20 kg/15 kg) & 8 Jumping Chest-to-Bar-Pull Up	55 Deadlifts (61 kg/43 kg) & 55 Wall-ball Shot ^†^ (9 kg/4 kg) & Row (55 Calories) & 55 Handstand Release Push-up	Thruster (29 kg/20 kg) & Burpees

* Rx wall ball shot targets: Male—10 feet; Female—9 feet; ^†^ Scaled wall ball shot targets: Male & Female—9 feet; ° Rx weight for Wk2 squat cleans round Rd1: 61 kg/38 kg, Rd2: 83 kg/52 kg, Rd3: 102 kg/65 kg, Rd4: 124 kg/79 kg, Rd5: 142 kg/93 kg; ^◊^ Scaled weight for Wk2 squat cleans Rd1: 43 kg/25 kg, Rd2: 52 kg/34 kg, Rd3: 61 kg/43 kg, Rd4: 70 kg/52 kg, Rd5: 83 kg/61 kg.

**Table 3:** Individual Workout Intensities and Workout Durations.

	Week 1	Week 2	Week 3	Week 4	Week 5
Est. Max HR *	%MHR ^†^	%MHR ^1^ (Time ^‡^)	%MHR ^†^	%MHR ^†^	%MHR (Time ^‡^)
194	90.72 ^s^	87.11 (12.00)	88.14 ^s^	89.18 ^s^	91.24 (13.78)
182	95.05 ^s^	96.15 (4.00)	90.66	95.60	95.05 (18.38)
185	102.70 ^s^	102.16 (16.00)	97.30 ^s^	105.41	107.03 (26.98)
189	88.89	88.36 (8.00)	90.48	86.77	91.01 (13.47)
185	89.73	89.19 (8.00)	89.19	89.19	94.05 (14.47)
187	91.98	90.91 (8.00)	91.98	91.44	93.58 (18.38)
175	106.29 ^s^	105.71 (4.00)	107.43 ^s^	105.71	106.86 (19.93)
189	94.18	92.59 (8.00)	94.18	92.06	96.83 (15.25)
Average	94.94	94.02 (8.50)	93.67	94.42	96.96 (17.58)

* Maximal heart rate (MHR) was estimated using the Age-predicted formula (220 − Age) (Fox, 1973); ^†^ %MHR was calculated using the formula [(Ave. HR/MHR) × 100]; **^‡^** Time was standardized for each of the weeks as follows: Week 1 = 20 min, Week 3 = 7 min, Week 4 = 14 min.; ^1^ Week 2 was standardized in 4-min rounds for all athletes; ^S^ indicates participant Scaled workout.

**Table 4:** Baseline and Weekly PRE-Comparisons (mean (*M*) ± standard deviation (*SD*))*.*

	*M* (*SD*)	*M* (*SD*)	*M* (*SD*)	*M* (*SD*)	*M* (*SD*)	*M* (*SD*)
**Energy Index**	3.63 (8.00)	10.88 (3.36)	9.13 (6.42)	6.88 (6.83)	6.50 (6.87)	9.75 (7.54)
**TMD**	109.63 (16.53)	104.00 (10.03)	104.25 (15.21)	106.25 (10.98)	105.25 (14.11)	102.00 (7.96)
**Vigor**	9.75 (4.37)	12.13 (2.95)	11.13 (6.40)	8.75 (6.65)	8.75 (6.56)	10.50 (7.09)
**Fatigue**	6.13 (6.38)	1.25 (2.55)	2.00 (4.47)	1.88 (2.36)	2.25 (5.26)	0.75 (1.04)
**Anger**	1.25 (2.12)	1.50 (1.31)	2.00 (3.51)	2.13 (2.90)	1.00 (1.60)	1.13 (2.10)
**Depression**	2.25 (3.28)	0.63 (0.92)	0.38 (0.74)	1.88 (4.55)	0.38 (0.74)	0.63 (1.41)
**Confusion**	3.13 (2.42)	1.75 (1.28)	1.75 (2.19)	1.13 (1.25)	2.25 (3.81)	1.25 (1.58)
**Tension**	6.63 (5.55)	11.00 (6.70)	9.25 (5.70)	8.00 (6.55)	8.13 (5.77)	8.75 (6.41)

**Table 5:** Mood Changes Across the 5-Weeks of the Competition (mean ± standard deviation).

	Week 1	Week 2	Week 3	Week 4	Week 5	*p*-Value (Week)
**Energy Index**						
PRE	10.88 ± 3.36	9.13 ± 6.42	6.88 ± 6.83	6.50 ± 6.87	9.75 ± 7.54	0.448
IP	3.38 ± 6.50 °	8.38 ± 6.91	8.38 ± 6.48	3.50 ± 9.86	1.00 ± 11.24	0.097
30P	10.00 ± 6.16	10.38 ± 10.00	12.25 ± 3.58	8.75 ± 6.20	4.00 ± 5.42 ^†^	0.022
60P	14.75 ± 8.66	10.75 ± 5.39	11.63 ± 4.00	9.38 ± 7.50 *	6.00 ± 5.40 * ^†^	0.010
*p*-value (time)	0.004	0.663	0.240	0.101	0.132	
**Total Mood Disturbance**						
PRE	104.00 ± 10.03	104.25 ± 15.21	106.25 ± 10.98	105.25 ± 14.11	102.00 ± 7.96	0.896
IP	112.13 ± 10.64 °	101.75 ± 11.02	107.25 ± 15.97	110.25 ± 18.99	117.00 ± 20.30	0.073
30P	99.13 ± 13.36 °	96.63 ± 14.61	95.75 ± 7.44 °	99.00 ± 15.62 °	105.5 ± 11.02	0.066
60P	91.88 ± 11.22 °	95.50 ± 8.07	97.00 ± 7.29 °	97.13 ± 12.08 °	101.00 ± 5.45	0.088
*p*-value	<0.001	0.040	0.008	0.001	0.043	
**Vigor**						
PRE	12.13 ± 2.95	11.13 ± 6.40	8.75 ± 6.65	8.75 ± 6.56	10.50 ± 7.09	0.403
IP	13.63 ± 8.21	14.63 ± 5.07	12.75 ± 5.39	12.63 ± 8.52	11.38 ± 12.09	0.795
30P	15.38 ± 4.66	15.00 ± 5.42	14.25 ± 3.92	14.13 ± 5.72 °	10.50 ± 8.62	0.154
60P	17.88 ± 7.72	14.88 ± 5.28	13.50 ± 4.72	14.00 ± 7.27 °	11.63 ± 9.16 *	0.037
*p*-value	0.085	0.261	0.107	0.040	0.861	
**Fatigue**						
PRE	1.25 ± 2.55	2.00 ± 4.47	1.88 ± 2.36	2.25 ± 5.26	0.75 ± 1.04	0.559
IP	10.25 ± 4.03 ^†^	6.25 ± 7.09	4.38 ± 5.21	9.13 ± 5.87 ^†^ °	10.38 ± 7.29 ^†^ °	0.005
30P	5.38 ± 6.57	4.63 ± 7.42	2.00 ± 2.78	5.38 ± 6.48 °	6.50 ± 5.95 °	0.062
60P	3.13 ± 5.17	4.13 ± 7.28	1.88 ± 2.70	4.63 ± 5.68 °	5.63 ± 4.95 °	0.155
*p*-value	< 0.001	0.074	0.095	< 0.001	0.004	
**Tension**						
PRE	11.00 ± 6.70	9.25 ± 5.70	8.00 ± 6.55	8.13 ± 5.77	8.75 ± 6.41	0.353
IP	9.38 ± 4.75	6.75 ± 5.63 °	7.50 ± 6.30	8.50 ± 7.63	8.75 ± 6.90	0.277
30P	5.13 ± 5.11 °	4.63 ± 3.74 °	4.63 ± 4.24 °	4.88 ± 6.38 °	5.00 ± 4.72	0.973
60P	3.50 ± 2.56 °	4.38 ± 2.83 °	5.25 ± 5.31 °	4.13 ± 3.52 °	4.25 ± 3.62 °	0.364
*p*-value	< 0.001	0.001	0.001	< 0.001	0.033	

* Indicates significant difference when compared to week 1 values at *p* < 0.05; ^†^ indicates significant difference when compared to week 3 values at *p* < 0.05; ° indicates significant difference from PRE at *p* < 0.05.
